# Design, fabrication, and optical characterization of one-dimensional photonic crystals based on porous silicon assisted by *in-situ* photoacoustics

**DOI:** 10.1038/s41598-019-51200-1

**Published:** 2019-10-14

**Authors:** Cristian Felipe Ramirez-Gutierrez, Harol David Martinez-Hernandez, Ivan Alonso Lujan-Cabrera, Mario Enrique Rodriguez-García

**Affiliations:** 1 Posgrado en Ciencia e Ingenieŕıa de Materiales, Centro de Física Aplicada y Tecnología Avanzada, Universidad Nacional Autónoma de México Campus Juriquilla, C.P. 76230 Qro., Mexico; 2 Programa de Física, Facultad de Ciencias Básicas y Tecnologías, Universidad del Quindío, Quindío, C.P. 630004 Colombia; 3 Ingeniería Física, Facultad de Ingeniería, Universidad Autónoma de Querétaro, C.P., 76010 Querétaro Qro., Mexico; 4 Departamento de Nanotecnología, Centro de Física Aplicada y Tecnología Avanzada, Universidad Nacional Autónoma de México Campus Juriquilla, C.P. 76230 Qro., Mexico

**Keywords:** Photoacoustics, Photonic crystals

## Abstract

We present a methodology to fabricate one-dimensional porous silicon (PSi) photonic crystals in the visible range by controlled etching and monitored by photoacoustics. Photoacoustic can record *in-situ* information about changes in the optical path and chemical reaction as well as in temperature, refractive index, and roughness during porous layers formation. Radiometry imaging can determine the carrier distribution of c-Si substrate that is a fundamental parameter to obtain high-quality PSi films. An electrochemical cell was calibrated through a series of single PSi layers that allows knowing the PA amplitude period, porosity, and roughness as a function of the current density. Optical properties of single layers were determined using the reflectance response in the UV-Vis range to solve the inverse problem through genetic algorithms. PhC structures were designed using the transfer matrix method and effective media approximation.Based on the growth kinetics of PSi single layers, those structures were fabricated by electrochemical etching monitored and controlled by *in-situ* photoacoustics.

## Introduction

Nowadays, groundbreaking sensors are based on photonic crystals (PhC)^1^, porous materials^2^, and bio-inspired structures that allow accurate and reliable measures through its optical and electrical response^3–6^. Moreover, PhCs are fundamental components of other optoelectronic devices such as light emitting diodes (LEDs) and lasers. One of the promissory materials for these applications is porous silicon (PSi) thin films. PSi is a nanostructured and nanocomposite material with diverse porous morphology, different surface chemistry, and the enormous surface area, commonly obtained through electrochemical etching in hydrofluoric based (HF) aqueous media. The self-limited character of the PSi electrochemical reaction allows the fabrication of homogeneous films and heterostructures^7,8^. Besides, it is possible to custom PSi properties by changing the growing parameters, oxidation grade^9,10^, and surface chemistry through functionalization^7,11,12^. This fact makes the PSi an excellent candidate to develop optical devices such as porous distributed Bragg reflector (DBR) and optical microcavities (OMC)^13,14^. However, the physicochemical properties of PSi are critically dependent on the etching parameters, and there are not theoretical models to predict the refractive index, absorption coefficient, thickness, porosity, and interfaces roughness. Nonetheless, *in-situ* methodologies based on infrared spectroscopy^15^, laser interferometry^16–18^, and photoacoustic^18–20^ have been developed as an alternative to monitor the formation in real time of PSi thin films that allows feedback to control the electrochemical reaction.

There are several reports about DBRs based on PSi and methods to fabricate tunable PSi thin films^21–25^. Vincent^21^ manufactured DBR in the infrared range applying a square waveform of current density to produce a periodic structure of the PSi layer. These current profiles produced an interleaved high (*η*_*H*_) and low (*η*_*L*_) refractive index.

Some limitations were found related to the thickness of the layers, bandwidth, spectral position, and optical quality. Pavesi *et al*.^26^ used the same methodology^21^ but changed the etching time of the high refractive index film randomly. This means that the obtained DBR is, in fact, a random system. Setzu *et al*.^27^ studied the optical properties of multilayered PSi system; they fabricated DBR using square waveform of current density selected arbitrary, and presented a methodology to control the interface roughness to improve the optical quality of PSi structures. Other works relate to applications of PSi DBR as chemical and biological sensors^28–30^, and mirrors for photovoltaics devices^31,32^. Nevertheless, a common characteristic in the cited works is that the fabrication of the PSi DBRs always depends in using an arbitrary value of current density and etching time, resulting in a random optical response referred to the position of photonic band-gap, limited bandwidths, and low optical quality. This means that before producing the PSi DBR there was not a previous design or control. Therefore, it is imperative to mention that random devices are not related to quasi-periodic or disordered photonic structures^33–35^. The randomness is referred to as no certainty of optical properties at determined experimental fabrication conditions. For these reasons, the *in-situ* monitoring of the fabrication processes is needed to obtain reliable PSi devices. Therefore, intrinsic and extrinsic parameters must be taken into account to produce high quality and reproducible PSi devices. Extrinsic parameters are closely related to the fabrication methods of PSi films to obtain a tunable thicknesses, reflective index (porosity), and smooth interfaces. On the other hand, intrinsic parameters are associated with the substrate quality which is directly related to the carrier distribution, the crystalline quality, and the optothermal surface stability^36–38^.

In this sense, there are few works about techniques that monitor the formation in real time of PSi films. Particularly interferometry and photoacoustic are non-contact and non-destructive techniques that can follow the etching rate, the evolution of thickness, porosity, and interfaces roughness in real time. However, the main limitation of interferometry is related to the monitoring of temperature during the chemical reaction; this is a crucial parameter because it changes the etching kinetics. As a solution for this limitation, this group has reported a differential photoacoustic system as well as a systematic study on following the PSi films formation^19,20^. The photoacoustic (PA) signal contains the information about the electrochemical reaction^20^, optical, and thermal properties of layered systems^39–41^. Indeed, photoacoustic is an excellent technique to follow the fabrication process and reach tunable optical devices based on PSi. Therefore, this work is focused on establishing a methodology based on photoacoustic to monitor the DBR fabrication and a procedure to design and customize optical devices based on PSi such as DBRs and OMC. This means, the design and fabrication of PSi photonic structures at defined wavelength ranges.

According to this, this work shows key-points such as substrate quality, etching rate, porosity determination, and a model to determine the refractive index of porous media by using photoacoustic and effective medium approximation (EMA)^42–44^ that are necessary to fabricate high-quality DBRs and OMC. Fabrication of tunable PSi thin films requires a previous determination of c-Si substrate quality. This can be done by using photocarrier radiometry spectroscopy (PCR) imaging. PCR is a non-contact, non-intrusive, and non-destructive technique that has been used for mapping the implant dose across the c-Si wafers^45–47^ as well as to determine the carrier distribution in p and n Si wafers^48^.

**Figure 1:**
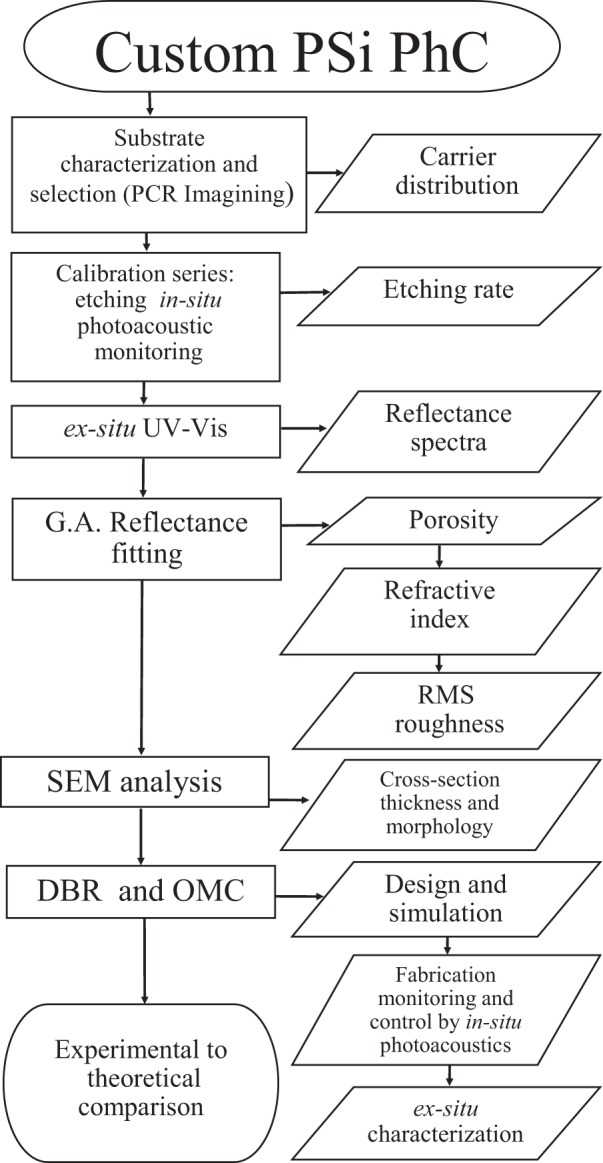
Flowchart of the procedures to design and fabricate PSi photonic crystals.

Thus, calibration of the electrochemical setup was performed. Then, a specific design of the device configuration was carried out, and finally, the fabrication monitoring and control processes are described. Figure 1 summarizes all steps followed that combine simulation, design, and experimental.

## Results and Discussion

### Substrate quality determination by PCR imaging

**Figure 2:**
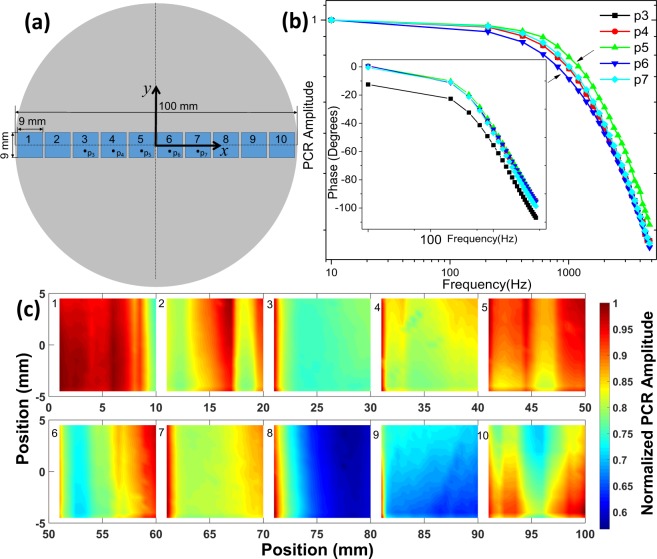
(**a**) Shows the silicon wafer and the scanned places by PCR. (**b**) PCR amplitude and phase of five points located at the center of the wafer. (**c**) Thermal images across the wafer. Laser beam radius 500 *µm*. Frequency 1 kHz.

It is well known that c-Si wafers present non-homogeneous carrier distribution^36,49^, and defects induced for the fabrications method and cleaving process. These can influence the PSi formation during the etching process given that the local magnitude of the electric field changes as a function of the position producing nonuniform porous nucleation. Usually, it is recommended to measure the nominal resistivity of the c-Si substrate^50^, but it is an average value that does not give information about local variations of the carrier distribution. Figure 2a shows ten different areas from the central part of the wafer that were used to obtain the photocarrier images. Figure 2b shows the PCR amplitude for points 3 to 7 while the inset in this figure shows the changes in the phase for a point in these locations. The changes in the PCR amplitude around 1000 Hz have been associated with the changes in the minority carrier diffusion coefficient that are directly related to the carrier concentration^36,37^. It means that the wafer does not have a uniform carrier distribution. Moreover, the phase signal is not quite sensitive to the changes in the carrier distribution. Figure 2c shows the PCR amplitude of the ten regions across the wafer, red colors represent high carrier concentration while the blue ones can be associated with a decrease in the carrier concentration. These PCR images evidence the non-uniformity in the carrier distribution that has to affect any electrochemical process and porous distribution.

### Etch calibration by *in-situ* photoacoustics

S1 calibration series was made to satisfied those criteria stablished for PCR imaging and Van der Pauw methods. The criteria ask for substrates with homogeneous carrier distribution and similar nominal resistivity. Mainly, it is considered a good quality substrate the one whose normalized PCR amplitude does not change more than 10% of the mean value such as those found in samples 3, 4, 7, 8, or 9 in Fig. 2c. On the other hand, sample 10 does not meet PCR criterion, so it is not used for further procedures. To verify the reproducibility of PA cycles, a second calibration series (S2) was performed.

The substrates that satisfied these conditions were selected to fabricate the calibration series. S1 and S2 are individual layers of PSi that were obtained upon varying the anodizing current density from 5 to 60 mA/cm^2^. Each etching was monitored using a differential photoacoustic system^18^, and temperature of the cell was kept at 25 °C. These series of individual layers were used as a calibration of the cell to obtain the of porosity, etching rate, photoacoustic cycle time, and interface roughness as a function of current density.

This technique allows controlling the etching time through the monitoring of PA cycles. In a previous publication of this group^19^, we have proved how to estimate the porosity using the PA amplitude and the sample thickness measured by SEM. PA effect is produced by the absorption of modulated light (heat source) that produces a heat diffusion process. Moreover, the changes in the optical path, as a result of the formation of the porous film, make a self-modulation of the intensity of incident radiation (changes in the reflectance) that modules the PA effect. This modulation is periodic^19^ and depends on the phase given for normal incidence by:

1 $$\delta (t,p)=\frac{2\pi {\eta }_{i}(p){d}_{i}(t)}{\lambda },$$

where *η*_*i*_(*p*) is the real part of the refractive index of the film that is porosity (*p*) dependent, *d*_*i*_ is the thickness film, and *λ* is the wavelength of the laser. The maximum of PA signal occurs when the reflectance of all the structure is a minimum, and it takes

place when:

2 $$\delta (t,p)=(m-\frac{1}{2})\pi ,\,{\rm{a}}{\rm{n}}{\rm{t}}{\rm{i}}-{\rm{r}}{\rm{e}}{\rm{f}}{\rm{l}}{\rm{e}}{\rm{c}}{\rm{t}}{\rm{i}}{\rm{v}}{\rm{e}}\,{\rm{c}}{\rm{o}}{\rm{n}}{\rm{d}}{\rm{i}}{\rm{t}}{\rm{i}}{\rm{o}}{\rm{n}}.$$

Consequently, the minimum of the PA signal is reached in a maximum of reflectance when:

3 $$\delta (t,p)=m\pi ,{\rm{r}}{\rm{e}}{\rm{f}}{\rm{l}}{\rm{e}}{\rm{c}}{\rm{t}}{\rm{i}}{\rm{v}}{\rm{e}}\,{\rm{c}}{\rm{o}}{\rm{n}}{\rm{d}}{\rm{i}}{\rm{t}}{\rm{i}}{\rm{o}}{\rm{n}}$$

for *m* = 1, 2, 3..., that is related to the number of PA cycles. These conditions allow to determine the refractive index for *λ*_0_ that is related to the porosity of the PSi film, if the thickness is known and PA amplitude signal ends in a local maximum or minimum (Eqs. 2 and 3).

**Figure 3:**
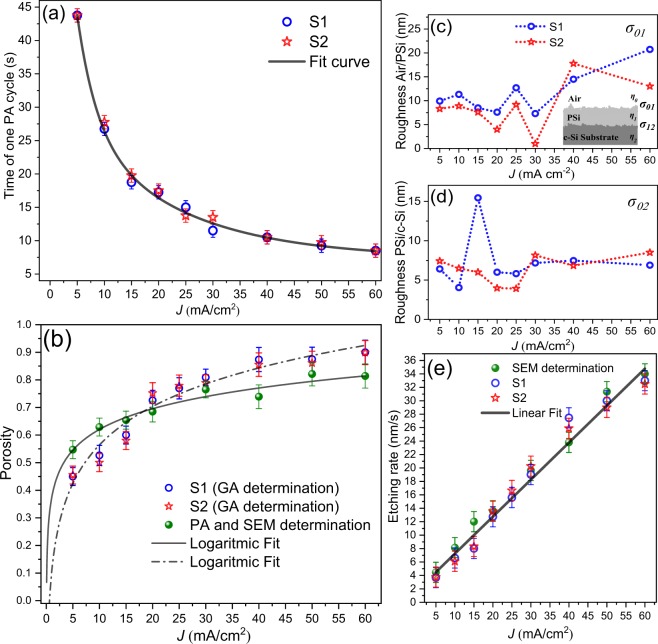
Calibration series parameters. (**a**) PA time as a function of current density that exhibits an exponential decay behavior. (**b**) Average porosity determined by GA and PA-SEM, (**c**) and (**d**) interface roughness as a function of current density determined by GA fitting of UV-Vis spectrum. (**e**) Etching rate that exhibits linear behavior.

Figure 3a shows the time of one PA cycle as a function of current density. As Eq. 1 shows, the increase of current density rises the etching rate and film porosity producing a reduction of the PA cycle. After that, reflectance spectra were measured and fitted by using genetic algorithms (GA) reported by Ramirez-Gutierrez^51–53^ to determine simultaneously the effective porosity (Fig. 3b), interface roughness^54^, and thickness using the Transfer Matrix Method (TMM).

Figures 3c,d show the interface roughness of air/PSi (*σ*_01_) and PSi/Si substrate (*σ*_12_) interfaces. This parameter is critically dependent on electrolyte composition and temperature. Only roughness values of less than 20 nm were found.

**Figure 4:**
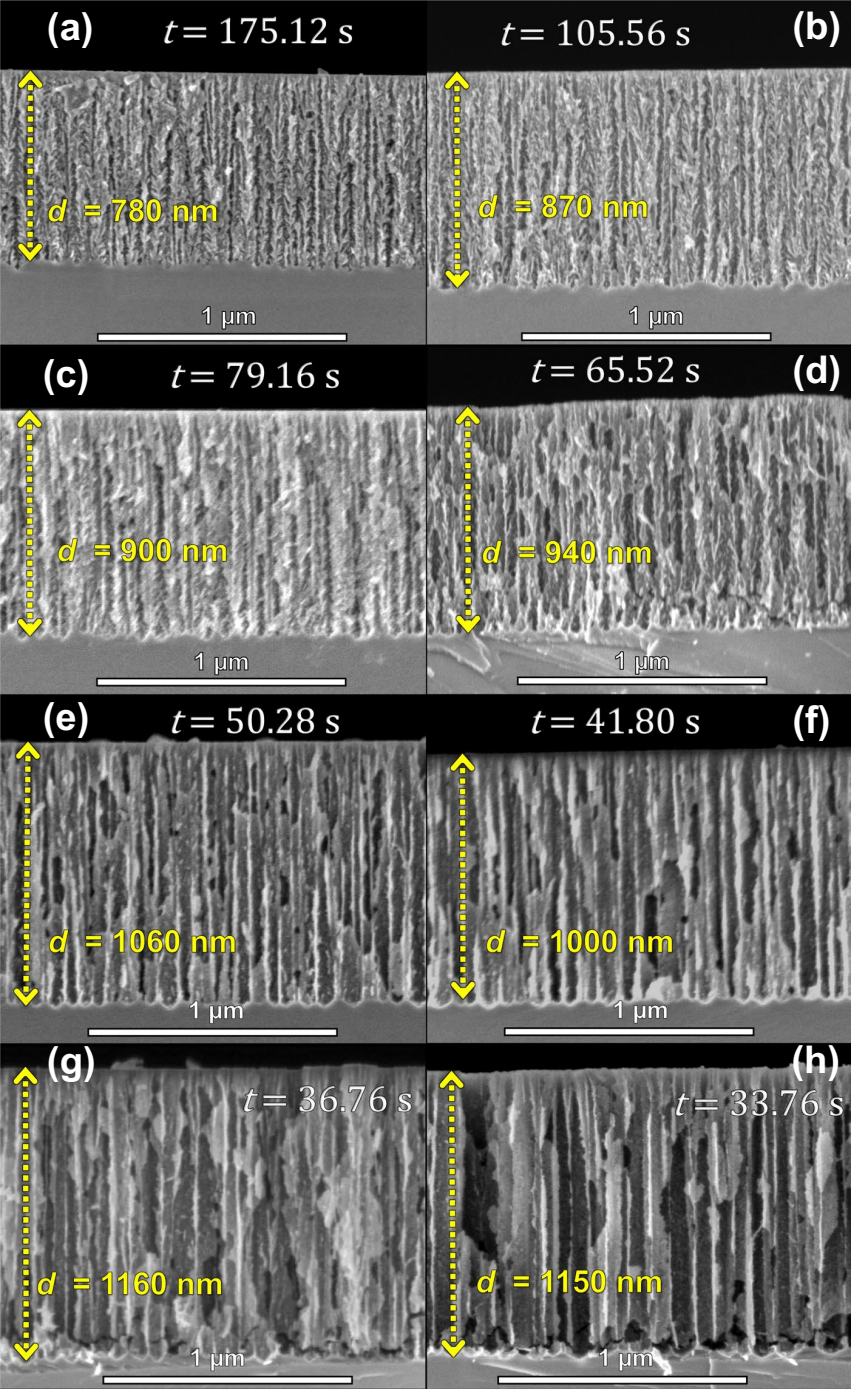
Cross-section SEM images of calibration series. (**a**) 5 mA/cm^2^, (**b**) 10 mA/cm^2^, (**c**) 15 mA/cm^2^, (**d**) 20 mA/cm^2^, (**e**) 30 mA/cm^2^, (**f**) 40 mA/cm^2^, (**g**) 50 mA/cm^2^ and (**h**) 60 mA/cm^2^. The images show the total anodizing time and thickness of each sample.

Porosity is usually determined by gravimetric analysis^55^, but in this work, two methods to determine it as a function of current density were used (Fig. 3b). In the first one, the reflectance spectrum of every single film was fitted and compared with a simulated one using the refractive index calculated by Landau-Lifshitz-Looyenga (LLL) EMA rule^42–44^. The second was carried out using the anti-reflective condition on the PA signal (Eq. 2), and the thickness was determined by SEM. This shows that it is probable to obtain refractive index for *λ*_0_ using Eq. 1, making possible to introduce this value in the LLL EMA to calculate the porosity. In order to achieve this, the refractive index of HF as 1.157^56^ and ethanol 1.365 were used. There are some discrepancies in the porosity values (Fig. 3b). Nonetheless, it is well known that the porosity determined by optical methods is model dependent^31,57^. Besides, the roughness of PSi interfaces is critically reliant on electrolyte temperature and composition (HF/surfactant ratio)^27^. For the calibration series, the parameters electrolyte temperature and composition were retained constants. Moreover, layer thicknesses calculated by GA and the ones measured by SEM (Fig. 4) were close. Also, the etching rate was obtained using two methods: the PA methodology fitting the PA amplitude^20^ and by direct calculation using the total etching time and the SEM thickness.

Figure 3e shows the etching rate as a function of current density obtained through thickness obtained by GA and SEM divided by total anodizing time recorded by PA amplitude. As can be seen, the etching rate obtained by the two methods has a linear behavior from 5 to 60 mA/cm^2^.

Figure 4 shows the cross section of some single films of the calibration series. It was observed in these images that porosity and etching rate increase as a function of the current density. Besides, all samples exhibited straight porous formation and interfacial roughness mainly in the interface PSi/substrate.

### Design and simulation of photonic structures

The PSi based one-dimensional PhCs were designed using the quarter-wave condition^58^ (Eq. 5), in which the optical thickness of each layer should be equal to a one-quarter of the central resonance wavelength (*λ*_0_). Therefore, by controlling the refractive index and thickness of each layer is possible to design a customized PhC. Furthermore, it is worth noticing that the refractive index of c-Si has a plateau between 500 and 1450 nm, and it increases rapidly for values near to UV as the absorption coefficient does^59^. It means that it is recommendable to design PSi PhC in the plateau region. Thus, all photonic structures of this work were designed in the plateau refractive index region.

The PSi is a composite material, hence, its optical properties can be described as a mixture of dielectric functions (effective medium approximation), that in the case of PSi is a mixture of dielectric properties of c-Si host matrix (${\hat{\varepsilon }}_{M}$) with incrustations within it of another material that full the pores (${\hat{\varepsilon }}_{1}$) (some gas or liquid). The incrustations size with a dielectric function (${\hat{\varepsilon }}_{1}$) in the majority system (${\hat{\varepsilon }}_{M}$) must be comparable or less than the wavelength of the radiation that interacts with the medium. The LLL effective dielectric function^43,44^ can be described as:

4 $${\hat{\varepsilon }}_{ef}^{1/3}={\varepsilon }_{M}^{1/3}+p({\hat{\varepsilon }}_{1}^{1/3}\,-{\hat{\varepsilon }}_{M}^{1/3})$$

where *p* represents the porosity, ${\hat{\varepsilon }}_{1}$ corresponds to dielectric function of the incrustations, and ${\hat{\varepsilon }}_{M}$ represents the c-Si dielectric function. In particular, the porosification of c-Si reduces significantly the refractive index compared to the c-Si. This means that the values available of refractive index for PhC design are between the c-Si and the filling material (i.e. *η*_*S*_ = 3.675 and *η*_0_*≈* 1 at 800 nm)^59^.

**Figure 5:**
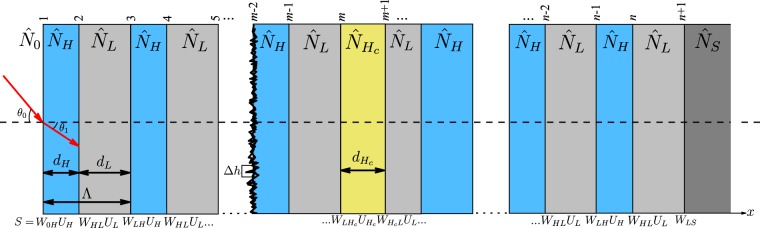
Schematic of the multilayer structure composed of *n* films that form *n* + 1 interfaces. The order of subscripts indicates the propagation direction.

For the DBRs and OMCs structures presented in this work, the reflectance is calculated through (TMM)^54,60^. As it is shown in Fig. 5, it is considered a stack of *n* alternating films with a high refractive index ${\hat{N}}_{H}={\eta }_{H}+i{\kappa }_{H}$ and low refractive index ${\hat{N}}_{L}={\eta }_{L}+i{\kappa }_{L}$ coupled to an incident media (air) with ${\hat{N}}_{0}={\eta }_{0}$, and a c-Si backing with ${\hat{N}}_{S}={\eta }_{S}+i{\kappa }_{S}$. This structure contains *n* + 1 interfaces, and Λ = *d*_*H*_ + *d*_*L*_ corresponds to the period of the structure. Each interface between two materials is represented by an admittance matrix **W**_*i*, *j*_ defined in terms of the optical admittance, which is a ratio of tangential components of the electric and magnetic fields amplitudes, and a phase matrix **U**_*m*_ that describes the action of the bulk. The product of all matrices resulting in the transfer matrix (**S**) of the stack. Besides, this formalism allows introducing the effects produced by random irregularities (roughness) in each interface through the modified Fresnel coefficients. This approximation supposes that the interface irregularities are much smaller than the incident wavelength, i.e., ∆*h λ*. Therefore, the description can be made by using the root mean square (RMS) roughness (*σ*_*k*_) of each interface. The effect of the interface roughness in the optical response of photonic crystal was studied by Lujan-Cabrera *et al*.^54^.

The DBR and OMC structures were designed fixing *λ*_0_ and the stopband width ∆*λ*. These parameters allow to obtain the thickness and refractive index of each layer by using the quarter-wave condition (Eq. 5).

5 $${\lambda }_{0}=2({\eta }_{H}{d}_{H}+{\eta }_{L}{d}_{L}),$$

To determine the stopband width was used the following expression:

6 $$\frac{\Delta \lambda }{{\lambda }_{0}}=\frac{\pi }{2}(\frac{1}{{\cos }^{-1}(\rho )}-\frac{1}{{\cos }^{-1}(\rho )}),$$

7 $$\rho =\frac{{\eta }_{H}-{\eta }_{L}}{{\eta }_{H}+{\eta }_{L}},$$

where *λ*_0_ is the central resonant wavelength or Bragg wavelength, *η*_*H*_ and *η*_*L*_ are the refractive indexes, *d*_*H*_ and *d*_*L*_ are the layer thickness (Fig. 5), and *ρ* is a refractive index ratio. The intensity, shape, and width of the stopband are also dependent on the number of periods of the structure. Considering these conditions (Eq. 5 and Eq. 7), refractive indexes are selected taking into account the desired bandwidth and stopband position, and these determine experimental parameters such as the current density and the etching time.

Three DBR prototypes were designed: the DBR550 is a structure designed near to the UV edge of plateau region with a stopband that does not contain the laser wavelength of PA setup. The DBR700 is a structure designed with a high refractive index contrast, this means a high contrast of layers porosity that produces a wide stopband. Also, PA laser wavelength is near to stopband in order to show the effect of the photonic bandgap formation over the PA signal. The DBR750 is a structure designed with a low refractive index contrast, this means a low contrast of layers porosity that produces a narrow-stopband. All DBRs are formed by 15 periods.

The OMC1 and OMC2 are PhC designed with the same conditions of DBR700 that includes a defective layer calculated by using the half-wavelength condition (*η*_*Hc*_ *d*_*c*_ = *λ*_0_*/*2), and different sequences were explored to avoid the absorption effect of the first pair of layers and to obtain high Q-factor cavities. Table 1 presents the constants for the design of each PhC and its characteristics such as structure sequence, resonant wavelength, and stopband width. These values were used to simulate the reflectance spectrum using the TMM.

**Table 1:** Constants for the design of the photonic PSi structures.

Structure	DBR550	DBR700	DBR750	OMC1	OMC2
sequence	(*HL*)^15^*S*	(*HL*)^15^*S*	(*HL*)^15^*S*	(*HL*)^3^*H*_*c*1_ (*HL*)^3^*S*	(*HL*)^3^*H*_*c*2_ (*LH*)^6^*S*
*λ*_0_(nm) (simulated)	550	700	750	700	700
∆*λ* (nm) (simulated)	78	164	66	311	235
*η*_*H*_	1.45	1.73	2.26	1.73	1.73
*η*_*L*_	1.16	1.20	1.97	1.20	1.20
*η*_*Lc*_	—	—	—	2.10	1.73
*d*_*H*_ (nm)	95	102	83	102	102
*d*_*L*_(nm)	131	146	96	146	146
*d*_*Hc*_ (nm)	—	—	—	330	167

### Fabrication and characterization of photonic structures

The first step was to select c-Si substrates with a homogeneous carrier distribution tested by PCR and Vander Pow method. This guaranteed the substrate quality. The refractive index, determined by the design (Table 1), fixes the porosity according to EMA theory^43,44^. At the same time, the porosity defines the current density for the etching process. These values were selected from the calibration series (Fig. 3b). The next step was to calculate the etching time using the value of the etching rate (Fig. 3d). Consequently, all the parameters mentioned above defined the photoacoustic profile that was used as a control parameter for the etching.

Table 2 shows the anodization time (*t*_*H*,*L*_) and current density (*J*_*H*,*L*_) for each layer of PhC, where the indices H and L indicate the high and the low refractive index layer respectively. Also, it shows the experimental values obtained for the layer porosity, thickness and roughness, and PhC resonance wavelength and stopband width. On the other hand, the PSi photonic structures present interfacial roughness inherently impacting in the optical quality. The roughness contribution at internal faces (H/L and L/H) has more impact on the optical quality than the first interface (Air/DBR) and the last one (DBR/substrate). Thus, Table 2 reports a roughness value of internal faces determined by GA and SEM. The previous works done by this group^18–20^ showed that the PA cycle observed during the PSi formation depends on refractive index, porosity, etching rate, and laser wavelength (Eq. 1), and the etching rate is almost constant for short anodizing times. Thus, each frequency component of PA signal is related to a single film formation. In order to obtain good control of the formation process, it was designed a selected layer thickness in which anodizing times were half multiplies of PA cycles. This means a minimum or a maximum to obtain symmetric cycles that is the case of samples DBR550 and DBR700. If the etching time does not satisfy this condition, the PA signal will have a beat behavior. Hence, the PA amplitude contains all frequency components of the etching rate of PSi films formation.

**Table 2:** Etching conditions and experimental parameters obtained for PSi PhCs.

Structure	DBR550	DBR700	DBR750	OMC1	OMC2
Sequence	(*HL*)^15^*S*	(*HL*)^15^*S*	(*HL*)^15^*S*	(*HL*)^3^*H*_*c*1_ (*HL*)^3^*S*	(*HL*)^3^*H*_*c*2_ (*LH*)^6^*S*
**Etching parameters**					
*t*_*H*_ ± 0.05 (s)	6.03	16.87	13.20	16.87	12.28
*t*_*L*_ ± 0.05 (s)	4.40	4.25	8.20	4.25	4.25
*t*_*Lc*_ 0.05 (s)	—	—	—	97.25	24.69
*J*_*H*_ (mA/cm^2^) ± 0.01	25	7.21	10	7.21	7.21
*J*_*L*_ (mA/cm^2^) ± 0.01	60	58.48	20	58.48	58.48
*J*_*Hc*_ (mA/cm^2^) ± 0.01	—	—	—	4.42	7.21
**Obtained parameters**					
*λ*_0_ (nm) (experimental)	572	725	764	700	707
∆*λ* (nm) (experimental)	64	140	67	293	232
*p*_*H*_ ± 0.01	0.75	0.68	0.57	0.68	0.68
*p*_*L*_ ± 0.01	0.92	0.91	0.70	0.91	0.91
*p*_*c*_ ± 0.01	—	—	—	0.55	0.68
*d*_*H*_ (nm)	90 ± 5	107 ± 8	110 ± 8	107 ± 6	100 ± 5
*d*_*L*_ (nm)	140 ± 9	143 ± 7	122 ± 8	143 ± 7	143 ± 7
*d*_*Hc*_ (nm)	—	—	—	335 ± 5	168 ± 7
^*σ*^ *HL*	15	20	12	20	20

The thickness of each layer represents an average values determined by SEM. The porosity and roughness were determined by GA. The spectral position and bandwidth were determined by the reflectance spectrum analysis.

**Figure 6:**
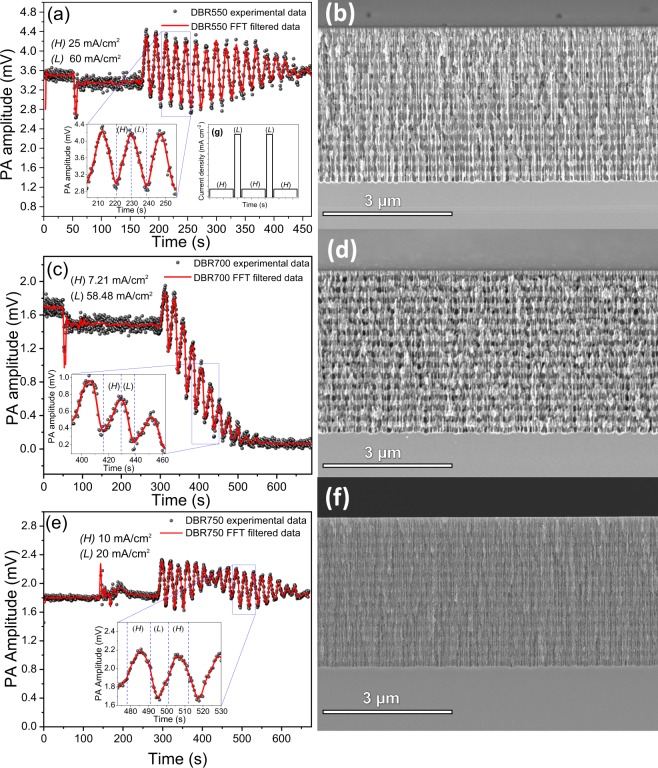
PA amplitude during DBRs fabrication. (**a**) DBR550, (**c**) DBR700, (**e**) DBR750. (**b**,**d**,**f**) are its corresponding cross-sectional SEM images. Inset (**g**) shows the current density profile used for DBRs fabrication. Insets in (**a**,**c**,**e**) correspond to a zoomed view of PA amplitude where dashed blue lines define each layer formation.

Figure 6 shows the *in-situ* PA signal for each designed DBRs as well as their cross-sectional SEM images. For the DBR550 (Fig. 6a) and DBR700 (Fig. 6c), the etching time was set as half of one PA cycle for the corresponding current density (see Table 2). Thus, a entire period in the PA signal represented a pair of *HL* layers. The porosity ratio between *H* and *L* allows clear identification of each layer as is shown in Fig. 6b,d. Furthermore, all interfaces exhibited roughness.

In the case of DBR550, the bandgap was centered at 550 nm and its bandwidth was 64 nm, so it did not reflect the wavelength (808 nm) of the laser that produces the PA effect. Therefore, the PA amplitude decreased only when the optical path increased. For DBR700 the PA amplitude decreased fast, even the PA periods disappeared after the formation of eleven pair of layers. This is an expected result given that the optical bandgap was near to the laser wavelength. This means that the most fraction of the incident radiation was reflected and the PA effect disappeared.

DBR750 PA signal in Fig. 6e corresponds to a beat profile since the etching time of each layer is not an integer multiple of a half PA period. For this reason, one period does not correspond to a pair of layers. However, PA amplitude frequency components allow the control of the etching process because the frequency associated with each layer is well known. DBR750 was designed with low porosity ratio and current densities that produce low contrast in the stack cross-sectional SEM image. Even though, the stack conformation is appreciable.

**Figure 7:**
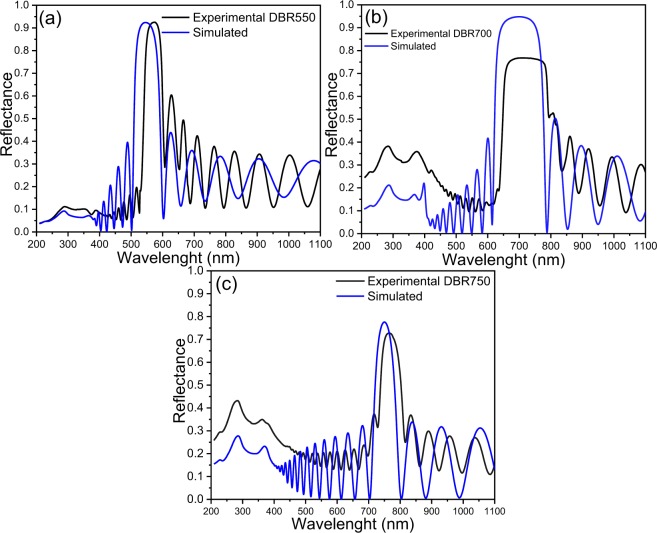
Reflectance spectra simulated (blue line) and measured (black line) for (**a**) DBR550, (**b**) DBR700, and (**c**) DBR750.

Measured and simulated reflectance spectra of DBRs are presented in Fig. 7, and the obtained structural and optical parameters are summarized in Table 2. All DBRs display redshifts and bandwidth narrowing compared with the simulated ones. These are due to parasite capacitance in the stack structure and the electrochemical circuit^61,62^ which the current source cannot control. Consequently, the discharge process held the etching during a short time generating layers thickness higher than the designed ones. Nonetheless, the deviation in the thickness of the layers, related to the designs, was around 15 nm that produced a redshift in the stopband position proportional to the optical path.

**Figure 8:**
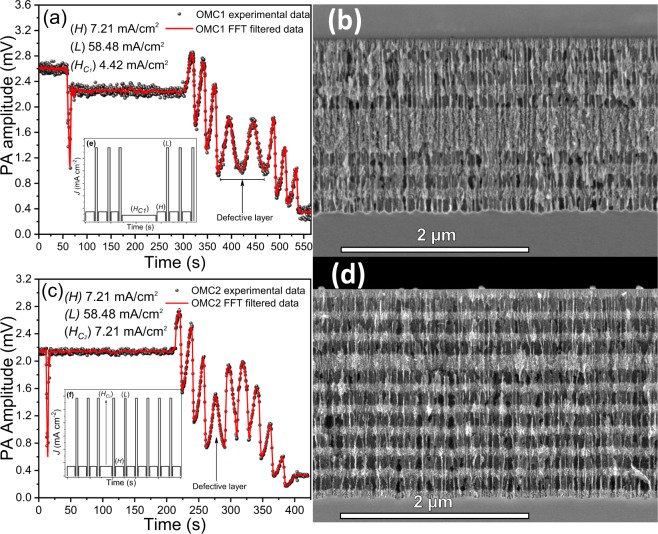
PA amplitude during OMC fabrication. (**a**) OMC1, (**c**) OMC2, and its respective SEM cross-sectional images (**b**,**d**). Insets (**e**,**f**) correspond to the current profiles used for OMC1 and OMC2 fabrication respectively.

Figure 8a,c shows the PA amplitude signals, and insets represent the current profile used to fabricate the OMCs. Figure 8b,d shows the cross-sectional SEM images for the OMCs. OMC1 and OMC2 were manufactured using the same conditions of DBR700, this means that it was introduced a defective layer into the DBR700 structure. The experimental parameters used for OMCs fabrication are summarized in Table 2. The OMC1 stack was designed as (*HL*)^3^*H*_*c*1_ (*HL*)^3^*S* sequence. In the micrograph (Fig. 8b), given that the current density used for the defective layer is near to the used one for *H* layer, there is no porosity contrast to differentiate *H* to *H*_*c*1_ layer, and it looks like a thick one. However, PA amplitude clearly shows the formation of a defective layer that corresponds to two PA cycles. The defective layer was designed with a thickness of 335 nm to make two cavities into the bandgap located at 650 and 787 nm. OMC2 (Fig. 8c,d was designed as (*HL*)^3^*H*_*c*2_ (*LH*)^6^*S* sequence with a defective layer of 168 nm that produces a microcavity (MC) at 668 nm. This sequence was selected to improve the reflectance percentage keeping the cavity depth. Clearly, the PA signal shows the formation of each *HL* pair of layers and the defective layer.

**Figure 9:**
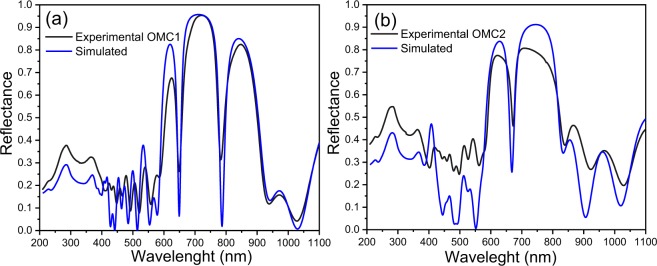
Reflectance spectra simulated (blue line) and measured (black line) of (**a**) OMC1 and (**b**) OMC2.

Likewise, in this stack, the sequence after the formation of defective layers is inverted, and it is appreciable in PA amplitude signal. In both OMCs the PA amplitude is sensitive to the photonic gap formation which is evidenced by the quickly signal attenuation. Figure 9 shows the reflectance spectrum of each OMC manufactured. Figure 9a corresponds to OMC1, and it is well appreciable the formation of two MC located at 649 and 783 nm with an FWHM about 27 and 32 nm respectively, that corresponds to a relatively high Q-factor. Shifts of 1 and 4 nm were produced for each MC compared with the simulation design, respectively. Fig. 9b corresponds to OMC2 and the MC is located at 672 nm, this means that a redshift of 4 nm was produced compared with the designed one. Moreover, the sequence of OMC2 improved the Q-factor whose FWHM is 16 nm. In both OMCs, a diminution is observed in the depth of the MC that is caused by the interface roughness of the stack^55^.

### Tolerance fabrication

The etching rate of c-Si in HF-based media was not constant for long etching times^18,20^, and the films presented porosity gradients as a function of depth^10,23^. Also, the electrolyte composition changed because of the chemical species released during the etching^7^. Furthermore, the silicon-electrolyte interface presented an inherent capacitance^61^ that could store enough charge to continue the etch even if the current supply was off. This effect produced layers thicker than the designed ones and generated a redshifted in the reflectance spectrum. This effect can be reduced if the current density decreases monotonously as a function of the time to compensate the remnant charges. Another alternative is to reduce the etching time. Nevertheless, in this work, and others related, PSi optical devices always presented deviations respect to the design ones. This is mainly attributed to the substrate quality and random fluctuations during the PSi formation. Hence, it is introduced the tolerance fabrication as a term related to the possible deviations of the optical response of the devices. In this work, we found a redshift in DRBs about 30 nm associated with layers ticker than the designed ones. In this case, the average reflectance shift was the layer thickness deviation times the refractive index. In the case of OMCs, the main effect was observed in the Q-factor that is affected by the interface roughness. Several works have reported PhCs based on PSi in the infrared region, which means that the thickness of the layers (optical path) is higher than those for PhC in the visible spectrum as is our case. Thus, deviations in layer thickness because of the intrinsic capacitance has more height on the optical response for the PhC designed in the visible region, usually a redshift.

## Conclusions

In this work was presented a complete methodology to design, fabricate, and characterize PSi one-dimensional PhC using *in-situ* photoacoustic as a monitor and control technique. The critical parameters that influence the PSi formation are the substrate quality, that is related to the carrier distribution along the wafer as was showed by photocarrier radiometry images as well as the control of the electrolyte composition and reaction temperature. The first step of this methodology consisted of the substrate quality determination to select pieces of c-Si with carrier homogeneity. The second step was the etching cell calibration by using photoacoustic that allowed knowing the behavior of manufacture parameters as a function of the current density to control the PSi formation. After that, simulation and design using the TMM and EMA formalisms were carried out to determine the manufacturing process of the customized devices. Finally, the fabrication was made with real-time monitoring and control using photoacoustic that can detect *in-situ* any deviation or unexpected event that could interfere in the formation process. The experimental results showed that electrochemical etching of c-Si in HF media is dependent on multiple parameters and each one plays a synergy role with others, i.e., the electrolyte composition and temperature in the interface roughness, carrier distribution and current density in the etching rate. Therefore, natural deviations in the morphology, such as layer thickness and porosity, produced variation in the optical response showed in the redshift compared with the ideal simulated device. SEM images and reflectance spectra showed clearly that this methodology allows the fabrication of reproducible DBRs and OMCs photonic devices with high accuracy. This methodology is a versatile technique that provides adequate real-time control in the optical path of multilayered systems that can be explored and implemented to monitor and control other thin films fabrication techniques such as vapor deposition, spin coating, or epitaxy.

## Methods

### Materials

Boron-doped monocrystalline c-Si wafers by Pure Wafer company with (*p*^++^) with 0.001 Ω cm of resistivity, (100) crystalline orientation, and single polish was used as a substrate. Deionized water, ammonium hydroxide solution (NH_4_OH wt 29%), and hydrogen peroxide (H_2_O_2_) supplied by Sigma-Aldrich were used for RCA cleaning process of c-Si substrates. Hydrofluoric acid (HF wt 48%) and ethanol (C_2_H_5_OH wt 99.98%) provided by Sigma-Aldrich were used for preparing the electrolyte used in this study. A platinum wire (99%) was used as a counter electrode. A total of 23 samples were prepared for these experiments.

### Sample Anodizing

**Figure 10:**
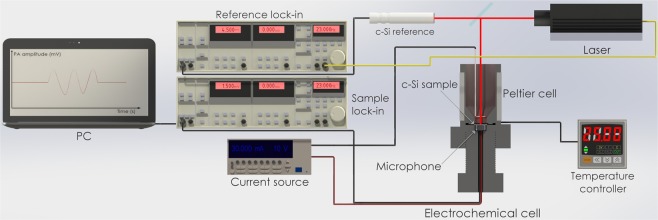
A stylized representation of the experimental electrochemical and photoacoustic setup used for PSi PhC fabrication. The surface samples were illuminated by a laser (808 nm) with square-wave modulated intensity. The Lock-In amplifiers and the current source were controlled using the GPIB card and software developed in Matlab (MathWorks, Inc.).

Figure 10 shows the etching and PA setup that consists of an electrochemical cell coupled to an open photoacoustic cell and an external (acoustic) cell as a reference. This configuration allows comparing the reference signal with the one obtained from the anodized sample, this reduces the noise from external sonic sources and improves the signal to noise ratio.

The PA setup is composed by a container that holds the electrolyte solution. A screw is used to set the microphone and the c-Si sample, and to seal the holder. An 808 nm laser line (<200 mW of power by Laser-Mate Group) modulated at 23 Hz is used as an excitation source. The laser was split by using a 60/40 beam splitter and the transmitted and reflected beams were focused on a c-Si external reference and on a c-Si sample respectively. Two electret condenser microphones were located at the back of c-Si samples; the microphones were polarized using a 9 V DC battery A Pt wire and a Cu ring were used as a counter electrode and as a collector respectively. The cell temperature was kept at 25^*◦*^C using Peltier cells regulated by a PID controller.

PSi was prepared by electrochemical etching in an electrolyte solution composed of HF and ethanol in a 3:7 volumetric ratio. The anodization current profiles were created using a precision Keithley current source (6220). During the etching, the photo-induced acoustic signals (amplitude and phase) were recorded using a Stanford Research SR830 Lock-In Amplifiers and GPIB-USB-HS (National Instruments) acquisition card. The cell was calibrated through two sample series (S1 and S2), each one composed by 9 samples anodized at 5, 10, 15, 20, 25, 30, 40, 50, and 60 mA/cm^2^. Three DBRs and two OMCs were anodized using current density values determined by the calibration series, and the etching time was monitored and controlled by the feedback of the PA amplitude. A total of 23 c-Si samples were used in the experiments.

### Sample characterization

The carrier distribution of c-Si substrates was characterized by using PCR implementing the methodology proposed by Mandelis and Rodriguez-Garcia^37,46,49^. An InGaAs infrared detector with spectral range 0.8–1.8 *µ*m, and a laser 532 nm was modulated from 10 to 5000 Hz. PCR images were taken at 1 kHz with a step size 100 *µ*m, and spot size of 40 *µ*m in the linear regimen^36^. Each image was taken in a 9 9 mm square and a 1 mm gap between areas.

Morphological characterization of PSi stacks was carried out with a high-resolution scanning electron microscope (Hitachi SU8230) employing secondary electron imaging operated at 1 kV. Reflectance measurements were conducted with a PerkinElmer Lambda 35 spectrophotometer in the near-specular (6°) configuration in a spectral range of 200–1100 nm.
